# Silent but Significant: A Case Report of Appendiceal Mucocele

**DOI:** 10.7759/cureus.66067

**Published:** 2024-08-03

**Authors:** Srinivasa Reddy, Darshana Tote, Anup Zade, Vasundara Gopalan, Shruthi Bikkumalla

**Affiliations:** 1 General Surgery, Jawaharlal Nehru Medical College, Datta Meghe Institute of Higher Education and Research, Wardha, IND; 2 General Surgery, Mahatma Gandhi Institute of Medical Sciences, Wardha, IND

**Keywords:** abdominal pain, inguinoscrotal swelling, mucocele of the appendix, acute appendicitis, surgical resection, appendiceal mucocele

## Abstract

Appendiceal mucocele is a rare disease that can sometimes mimic acute appendicitis or be discovered accidentally during surgeries. The clinical presentation of appendiceal mucocele is observed as lumen distension due to mucin accumulation. This condition has both benign and malignant underlying etiologies, which can be confirmed by histopathological examination. Acute presentation of appendiceal mucocele is rare and mostly resembles the symptoms of acute appendicitis. The treatment of appendiceal mucocele is crucial due to the risk of pseudomyxoma peritonei caused by the spread of mucus, mucocele perforation, or the presence of malignancy such as mucinous carcinoma. Surgical resection, either appendicectomy, typhlectomy, or sometimes right hemicolectomy, is the recommended management approach. This is a case of a 74-year-old male with pain in the abdomen as the major presenting complaint. He had a palpable right iliac fossa mass. The diagnosis of appendiceal mucocele was made by contrast-enhanced computed tomography, which was later confirmed by histopathology. The patient underwent surgical resection and was doing well at the three-month follow-up.

## Introduction

Appendiceal mucocele is a rare disease, often diagnosed incidentally. It is characterized by the dilatation of the appendix due to the accumulation of mucin secretion, irrespective of the underlying disease etiology. Its clinical symptoms might resemble acute appendicitis [1]. Clinical symptoms can include abdominal distension, abdominal mass, vomiting, weight loss, obstipation, and nausea [2]. The secretion of mucin has been reported to be of both neoplastic and non-neoplastic origins. Neoplastic causes include mucinous cystadenomas and mucinous cystadenocarcinoma, while common non-neoplastic reasons include mucosal hyperplasia, retention cysts, and obstructive mucoceles [2,3]. The incidence has been noted to be between 0.2% and 0.7%, with major subtypes such as mucinous cystadenocarcinoma, mucosal hyperplasia, retention cyst, and mucinous cystadenoma [2,4]. It is frequently observed in individuals over 50 years of age, with a female preponderance, though there are cases reported in young individuals [4,5].

Appendiceal mucoceles are typically diagnosed incidentally through radiological imaging and intraoperative examination, followed by confirmation through histopathological examination. Ultrasound screening can reveal a vermiform appendix and an onion-skin-like appearance. A preoperative diagnosis can aid the treatment strategy and help reduce the risk of intraoperative and postoperative complications [2,4,6]. Clinical diagnosis can be delayed or challenging due to vague presentations, such as lower abdominal pain and nausea, or those similar to acute appendicitis. Delayed diagnosis can lead to adverse outcomes, including spontaneous rupture of the appendix and spillage of mucinous contents into the peritoneal cavity, leading to pseudomyxoma peritonei [7]. Surgical removal of the appendix is recommended in patients without intestinal perforation. Surgical management of the mucocele of the appendix can be carried out by either an open-surgery approach or a laparoscopic approach, depending on the extent of disease spread, associated co-morbidities, and the surgeon’s analysis [2,6,7]. This case report discusses a 74-year-old male who presented with abdominal pain and a right iliac fossa mass. He was later diagnosed with appendiceal mucocele by computed tomography (CT) imaging preoperatively and managed successfully by exploratory laparotomy.

## Case presentation

**Figure 1:**
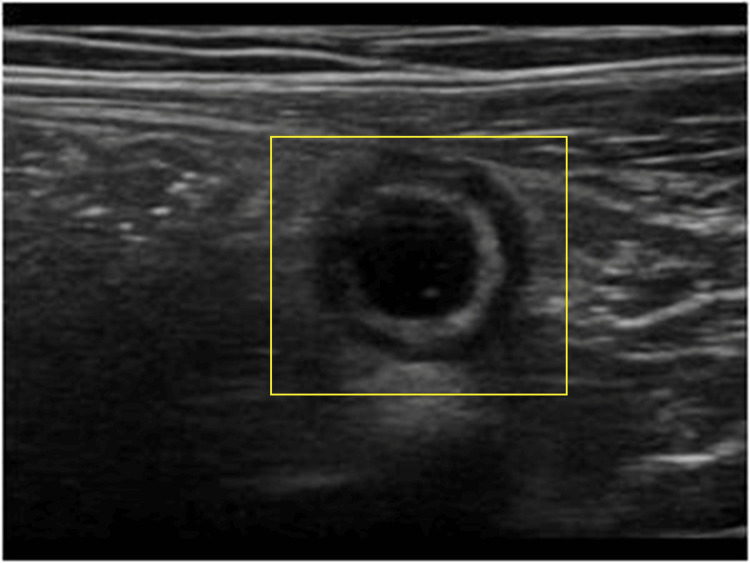
Ultrasound imaging of the abdomino-pelvic region Highlighted area indicates a heterogenous cystic mass lesion in the right iliac fossa

A 74-year-old male patient visited our hospital with major complaints of abdominal pain for two days and swelling over the right iliac fossa mass for two years. Physical examination showed a palpable mass in the right iliac fossa; the rest of the abdominal examination was normal. The patient was further subjected to ultrasound imaging of the abdomino-pelvis, which was suggestive of a large, heterogeneous cystic mass lesion in the right iliac fossa, which might be indicative of appendiceal mucocele (Figure 1).

The laboratory parameters of the patient are presented in Table 1.

**Table 1:** Lab parameters of the patient CEA: carcinoembryonic antigen; mm/hr: millimeter/hour; ng/mL: nanograms/milliliter

Parameter	Patient value	Normal range
Erythrocyte sedimentation rate	80 mm/hr	Male; >70 years: <30 mm/hr
Serum CEA	1.8 ng/mL	0 to 2.5 ng/mL (median level is 3.4 ng/mL in men)

**Figure 2:**
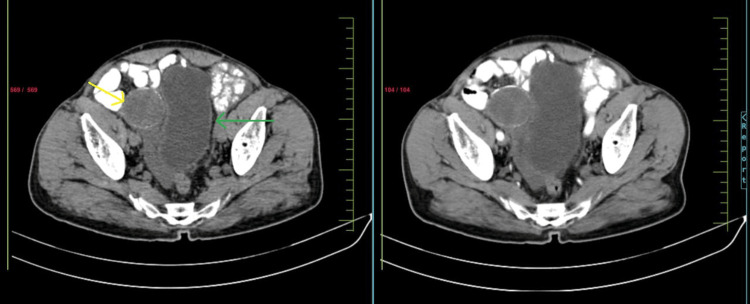
CECT abdomen-plain and arterial phase representing mucocele of the appendix (axial cut section) The yellow arrow shows mucocele and the green arrow indicates the bladder CECT: contrast-enhanced computed tomography

**Figure 3:**
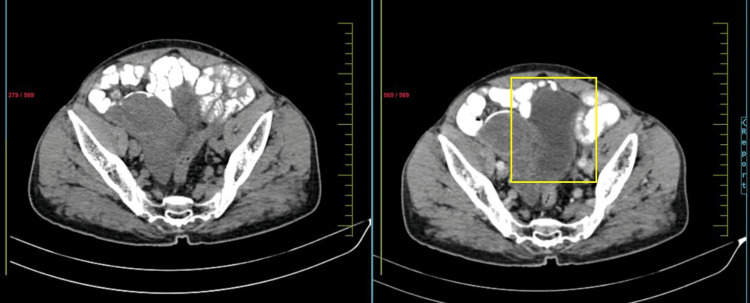
Computerized tomography image showing mucocele of appendix abutting the urinary bladder (plain and venous phase)

Contrast-enhanced computed tomography (CECT) of the abdominopelvic region revealed a lobulated hypodense lesion in the right iliac region, approximately 9 x 7 cm, medially abutting the urinary bladder and sigmoid colon, and laterally abutting the caecum and right internal iliac vessels, with a right inguinal hernia and encysted hydrocele of the cord (Figures 2-3).

**Figure 4:**
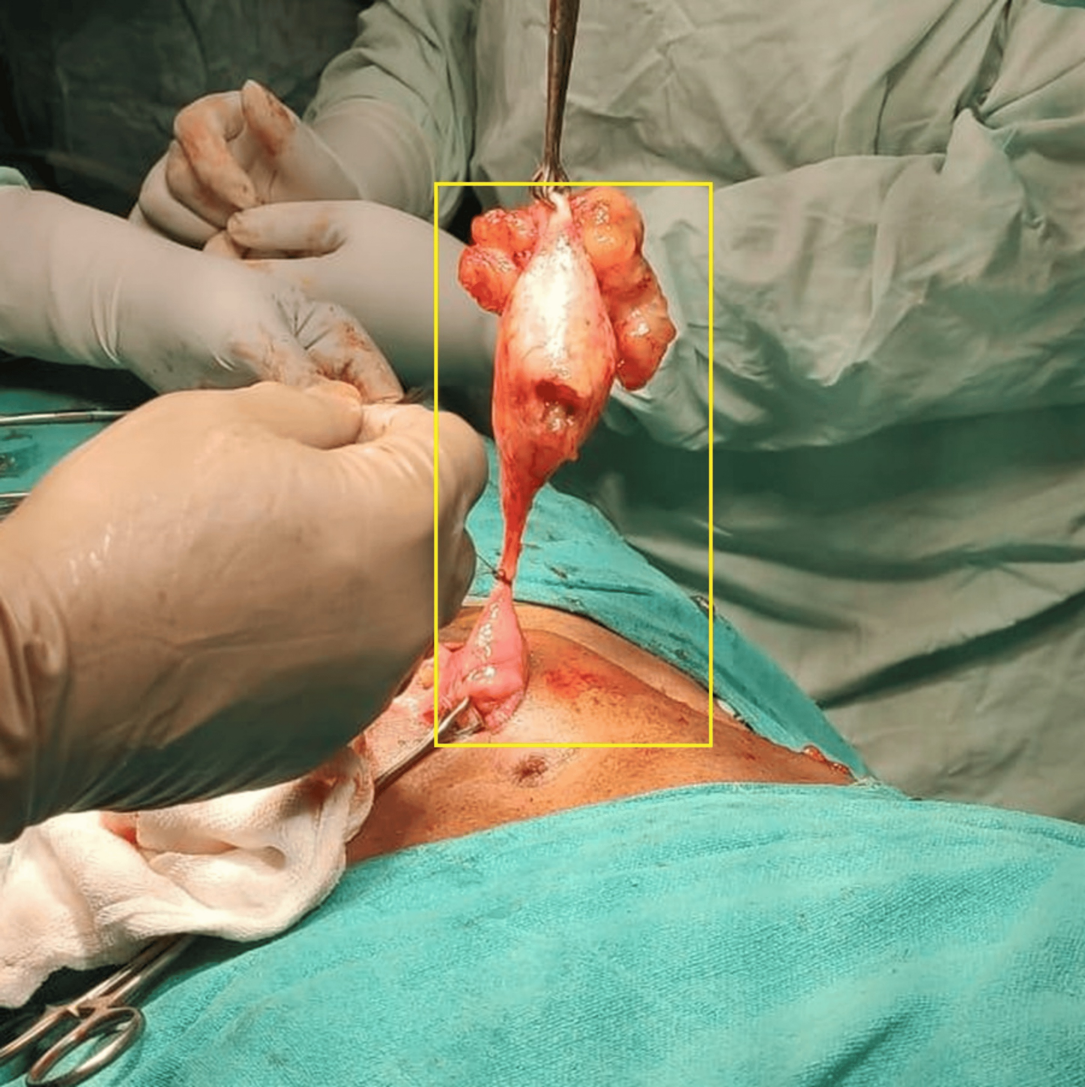
Intra-operative image of the patient

Carcinoembryonic antigen (CEA) was found to be normal; hence, the patient was surgically planned for exploratory laparotomy without right or left hemicolectomy. A perforated mucocele of approximately 7 x 4 cm was found intraoperatively. The mucocele involved the tip and body of the appendix, and the base of the mucocele appendix was free (about 2 cm from the caecum) (Figure 4).

**Figure 5:**
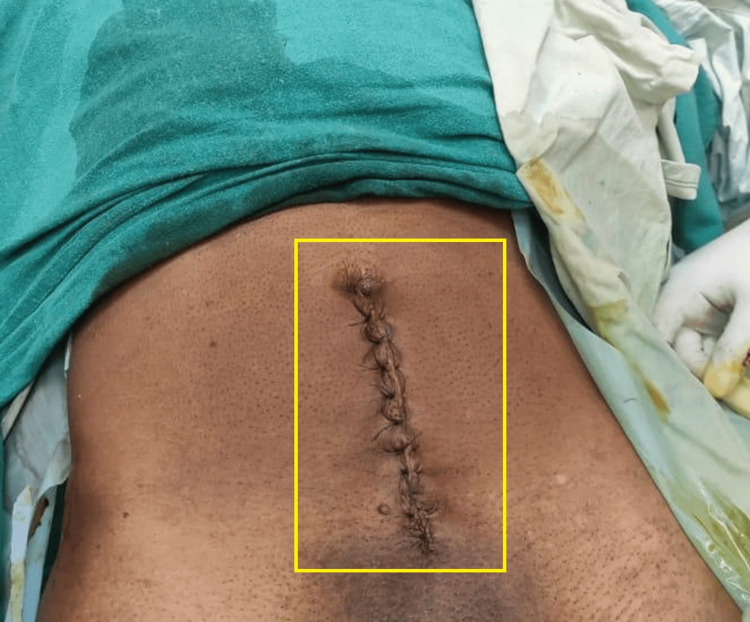
Immediate postoperative image of the patient

**Figure 6:**
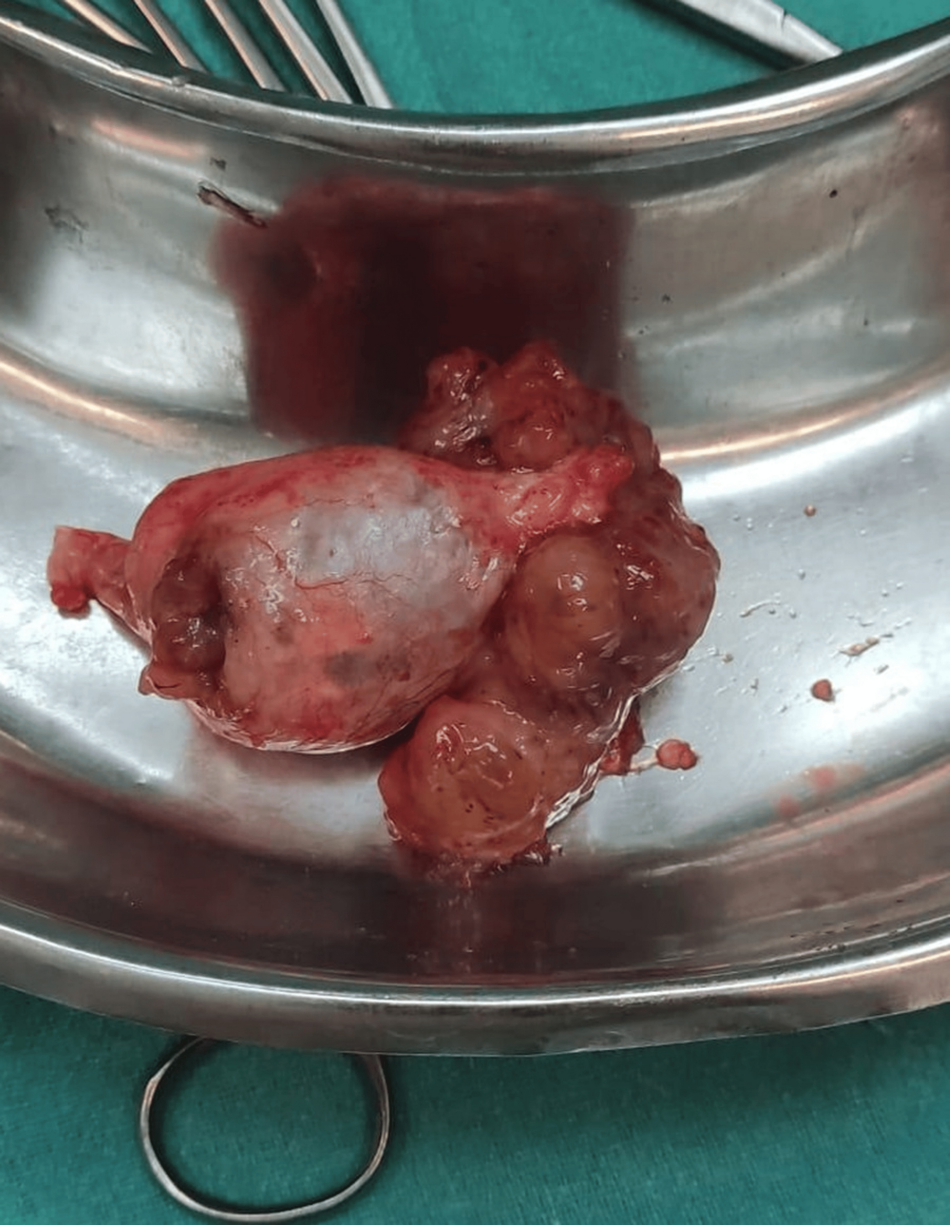
Excised specimen of the appendiceal mucocele

**Figure 7:**
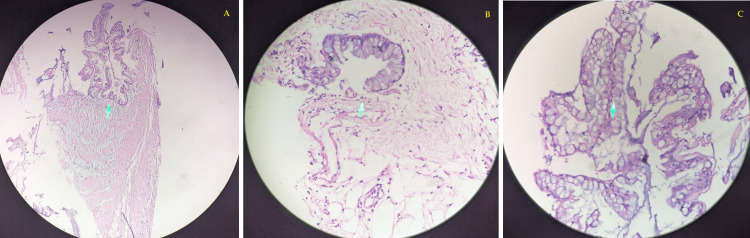
Histopathological slide of the excised sample A) 10x magnification; B) 40x magnification; C) 100x magnification

The patient was treated postsurgery with the following: injection of ceftriaxone + sulbactam 1.5 g intravenous/BD, injection of metronidazole 100 mL intravenous/TDS (three times a day) for five days, and injection of amikacin 500 mg intravenous single dose for three days. The excised specimen was sent intraoperatively for frozen section histopathological examination, which was suggestive of benign mucocele of the appendix (Figures 5-7).

There were no intraoperative complications. The patient was discharged after seven postoperative days and was noted to be recovering well without any postoperative complications.

## Discussion

Appendiceal mucocele is most commonly detected incidentally during the diagnostic work-up or intraoperatively in patients with associated comorbidities [1,2]. It might be asymptomatic in a few cases, in contrast to some patients where it is reported to be associated with lower quadrant abdominal pain, as noted in this case, who had right lower quadrant pain [4]. A palpable mass has also been observed in 50% of the patients [6]. A mucocele is a condition characterized by a mucus-filled appendix. It can be classified into two main categories: non-neoplastic and neoplastic. Non-neoplastic mucocele includes hyperplasia, where an increase in the number of cells causing a thickening of the appendiceal lining is observed, and retention cysts, are formed due to the retention of mucus. Neoplastic mucocele, according to the WHO classification, includes mucinous adenomas, which are benign tumors of the appendix that produce mucus; low-grade appendiceal mucinous neoplasms, which have low malignant potential and are characterized by mucus production; and mucinous adenocarcinomas, which are mucous-producing malignant tumors of the appendix [7]. Each type has distinct pathological features and implications for treatment and prognosis. On the basis of the type of mucocele, the treatment approach is determined by the condition of the appendix base and the presence of lesions in the cecum. A free base (up to 2.0 cm) is treated by appendectomy and lymphadenectomy, and histological analysis of the frozen section, if found benign, is followed up; in cases of any histopathological concern, further examination is done. In patients having a compromised base (>2.0 cm) or a lesion in the cecum, a typhlectomy is performed, and the excised section is sent for histopathological examination. Benign tumors are monitored, and for malignant tumors, right colectomy is performed [8].

This structured approach ensures that appropriate surgical and pathological evaluations are carried out to guide further treatment and follow-up. Appendiceal mucocele can be diagnosed with the help of simple radiological modalities such as ultrasound, which shows appendiceal mucocele as homogeneous hypoattenuating material with Hounsfield values similar to those filled with water. The diagnosis can be confirmed by CT. It can be differentiated from acute appendicitis by the absence of inflammatory markers [9,10]. A curvilinear mural calcification can be seen on CT imaging, but it is observed only in 50% of the cases. The presence of infection can be noted as the presence of air-fluid levels or intraluminal gas bubbles [9]. Colonoscopy can also be a useful tool in the diagnosis of appendiceal mucoceles, which show the characteristic volcano sign [11]. The patients with lower intra-abdominal pain can or cannot be related to the presence of mucin secretion or non-mucinous acute appendicitis. Surgical management by laparoscopic and open incision methods has been published, though management by laparoscopic methods has been associated with mucocele rupture and spillage of the mucin. Some of the commonly recommended approaches include appendectomy, cecal resection, right hemicolectomy, and debulking for the prevention of further complications [6,10]. Hence, the surgical approaches recommended for treating a mucocele of the appendix depend on several factors, including the integrity of the wall of the appendix, the dimensions of its base, and the histopathological examination of the cause of the mucocele [2,4,10,12]. Timely diagnosis has been associated with a good outcome; however, preoperative diagnosis is challenging due to its low incidence rates and vague clinical presentations [6,9,10]. Hence, this case can serve as an example of timely diagnosis and management of appendiceal mucocele, adding knowledge to the existing medical literature, which might help clinicians be vigilant in cases with similar symptoms and also be supportive in improving outcomes.

## Conclusions

In conclusion, a mucocele of the appendix is a rare condition that can be benign or malignant, and its diagnosis is crucial to prevent complications like peritoneal contamination and other severe issues. It can be a diagnostic challenge due to non-specific clinical symptoms. Benign appendiceal mucoceles can be managed by simpler methods, such as appendectomy or typhlectomy, based on the portion of the appendix involved. Cases with malignant diagnoses might require resection. The role of intraoperative frozen histopathology is crucial for pathological diagnosis and the decision on the extent of surgery. Although the majority of cases involve incidental findings of mucocele of the appendix, this case is reported as a successful example of preoperative diagnosis and management.
